# An Itch to Dilate: A Case Report of an Intralenticular Foreign Body in a 57-Year-Old Man

**DOI:** 10.7759/cureus.69702

**Published:** 2024-09-19

**Authors:** Tommy Bui, Anh Bui, Mark Manocha

**Affiliations:** 1 Ophthalmology, Southeast Campus of the Medical College of Georgia, St. Joseph's/Candler Health System, Savannah, USA; 2 Arts and Science, McMaster University, Hamilton, CAN; 3 Ophthalmology, Augusta University Medical College of Georgia, Savannah, USA

**Keywords:** dilation, intralenticular foreign body, intraocular inflammation, lensectomy, penetrating eye injury

## Abstract

A 57-year-old man presented with a foreign body sensation in the left eye after using a metal pellet air gun seven days prior. Following an unremarkable exam at urgent care, the patient was prescribed topical ointment and instructed to follow up with an ophthalmologist for continued problems. At ophthalmology, the patient reported decreased vision of 20/30 and pain with accommodation in the left eye. Intraocular pressure was 16 mmHg. A slit lamp examination of the left eye showed full thickness horizontal scarring superior to the visual axis with a negative Seidel sign. The iris was normal, and the anterior chamber was deep and quiet. There was a slight abnormal aberration of light in the lens on retro-illumination. After dilation, a large metallic foreign body inferiorly embedded in the anterior lens was noted, violating the anterior capsule. The foreign body was successfully removed using a magnetic probe. Following lens phacoemulsification and the removal of cortical material, the capsular bag was noted to be intact. An intraocular lens implant was placed and well-centered. The patient experienced no complications, and his vision improved to 20/20 the subsequent day. This case report demonstrates the need for high clinical suspicion of embedded foreign bodies in patients near high-speed projectiles and the importance of dilation to rule out the presence of intralenticular foreign bodies.

## Introduction

Ocular trauma is a significant cause of ocular morbidity in the working populations [1]. Penetrating eye injuries in workers frequently result from a lack of protective eyewear. Intralenticular foreign bodies (FBs) are rare in ophthalmic practice, usually presented with signs of inflammation, and diagnosed on slit lamp examination [2]. The rarity of FBs makes presentations with the lack of intraocular inflammation and foreign body visualization challenging to diagnose, but a good visual prognosis is likely with appropriate treatment [1]. The risk of ocular siderosis highlights the importance of proper diagnosis of metallic FBs and removal [3]. We describe a case of an intralenticular FB in a 57-year-old man, which remained undetectable until dilation revealed the location. In this report, the ability of the FB to hide behind the iris and the utility of dilation for visualization is of interest.

## Case presentation

**Figure 1:**
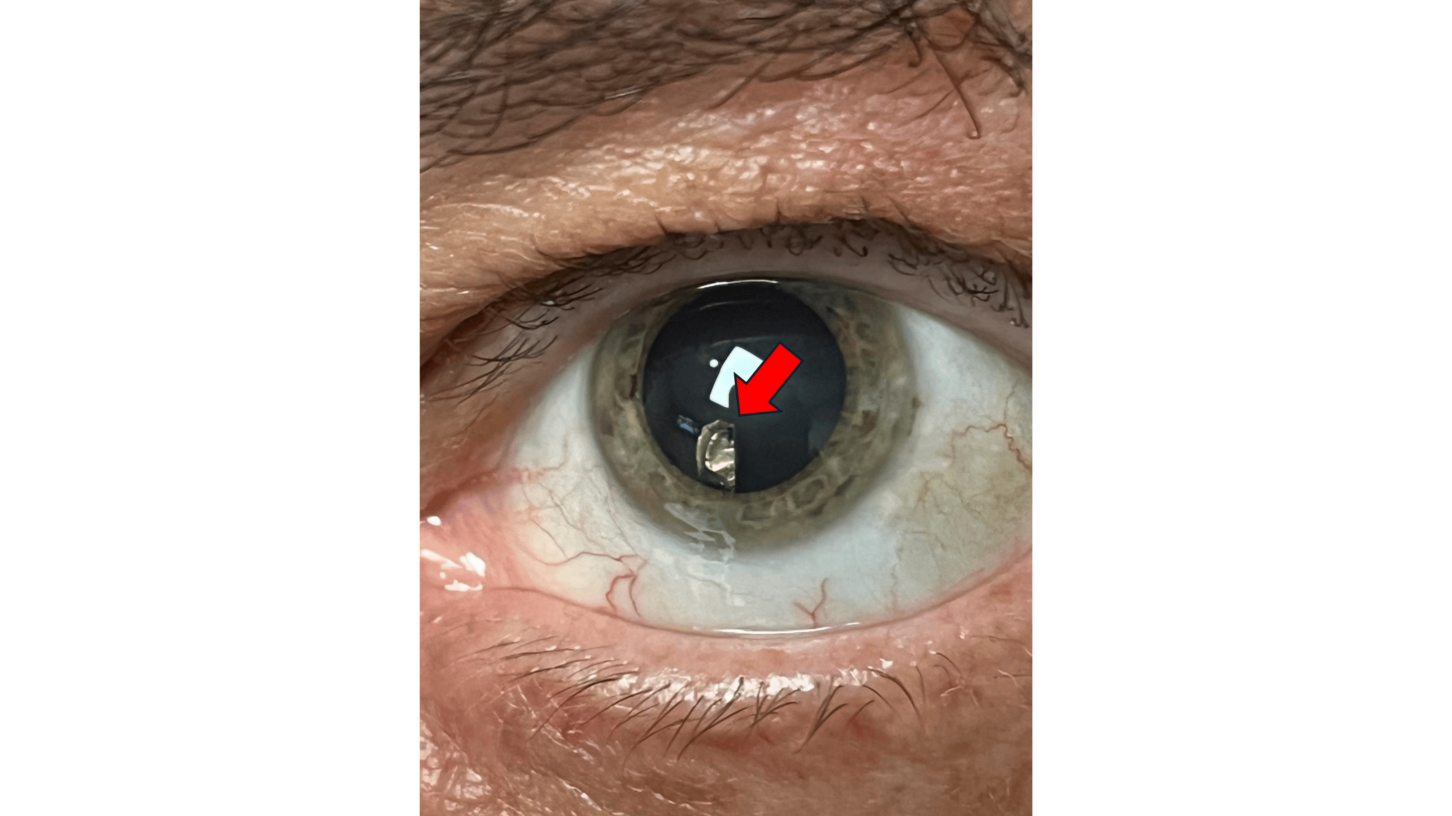
Before dilation, the foreign body was visually obscured by the iris. This post-dilation photo shows the location of the previously hidden metallic intralenticular foreign body (indicated by a red arrow).

A 57-year-old man presented with an FB sensation in the left eye (OS) for one week. He had no relevant past medical or ocular history, including no prior ocular surgeries or trauma. The patient was a car mechanic whose symptoms began after drilling into metal without eye protection at work. He had no recollection of any projectiles flying into his eyes or any trauma at work. He visited urgent care that day and was prescribed topical ointment after an unremarkable examination. The patient was told to follow up with an eye provider if any problems persisted. In the eye clinic three days later, the patient presented with persistent foreign body sensation, decreased vision, and pain with accommodation OS. Visual acuity was 20/20 OD and 20/30 OS. Slit lamp examination OS showed a full-thickness horizontal scarring superior to the visual axis with a negative Seidel sign and no intraocular inflammation. Intraocular pressure was 16 mmHg in both eyes (OU). The iris was normal OU, and the anterior chamber was deep and quiet OU. Slight abnormal aberration of light in the lens OS with retroillumination prompted dilation, which revealed a metallic intralenticular FB inferiorly. The intralenticular FB was embedded in the anterior lens, violating the anterior capsule (Figure 1). The patient was urgently scheduled for removal of the foreign body under general anesthesia. The eye was entered with a superior paracentesis and temporal main clear corneal incision. Intracameral moxifloxacin was administered. A capsulorhexis starting at break superior to the FB was created with particular attention to avoid radializing at the site of the anterior capsule violation. Hydrodissection and hydrodilineation with a balanced salt solution and a 27-gauge cannula were performed to separate the lenticular body and surrounding cortex from the capsule and epinucleus. With a magnetic probe, the intralenticular FB was engaged, separated from the surrounding cortical material, and removed through the main temporal incision. The patient underwent subsequent lens phacoemulsification. The posterior capsular bag was noted to be intact, and an intraocular lens implant was placed in the bag and well-centered. The patient’s vision OS improved to 20/20 the next day, and he experienced no intraocular complications. The patient was instructed to use moxifloxacin and prednisolone drops four times daily until the one-week follow-up.

## Discussion

The National Eye Trauma System Registry determined that fewer than 10% of patients wore eyewear or goggles during ocular injury [4]. A 2008 study reviewed 96 patients with metallic intraocular FBs and discovered a high frequency of magnetic FBs in 64 eyes (69%) [3]. These findings reiterate the importance of eye protection, especially when working with metals, which is paralleled in our report. Early surgical removal of FBs should not be delayed, especially with recent surgical advances that enable safe removal of FBs with good visual results [5]. Without proper diagnosis and treatment, ocular injuries associated with intralenticular FBs can result in severe health complications. A retained ferrous intraocular FB can cause iron deposition in ocular tissues, leading to siderosis bulbi [6]. This condition contains clinical features including iris heterochromia, pupillary mydriasis, vitritis, and pigmentary retinal degeneration [7]. Untreated intralenticular FBs following eye trauma can also cause endophthalmitis, cataract, and retinal detachments [8].

Though the good visual result is consistent with most documented cases [1], our case emphasizes mandatory full ophthalmic dilation with dilation if an intraocular foreign body is suspected. It also highlights that high suspicion is warranted in any patient experiencing ocular irritation after high-risk activities. The patient’s occupation and inciting event of ocular trauma raised suspicion for an underlying metallic FB, despite the absence of intraocular inflammation and initial concealment of the FB. The projectile's high speed and specific angle as it entered the eye created a Seidel-negative corneal wound without iris depigmentation or lens disruption, further complicating the clinical appearance [1]. The subtle light aberration was noted with retroillumination on slit eye examination, and after dilation, the metallic FB was discovered behind the iris. Since patient’s vision may suffer greatly from a delay in accurate diagnosis, it is critical to promptly visualize intralenticular FBs in cases of ocular trauma. In patients with symptoms of penetrating eye injury, the presence of an intraocular foreign body should not be ruled out, even if the patient denies this possibility [9]. Clinicians should be aware of the possibility of FBs hiding behind the iris and the crucial role of dilation in visualizing such instances. 

Dilation facilitates a more complete examination of the lens as the iris contracts to allow for visualization of FB embedded in a previously hidden part of the lens [5]. Further research is needed to clarify the utility of dilation in certain cases as opposed to other modalities, such as X-ray and CT scans, to confirm the presence of intralenticular FBs [9]. It should be noted that magnetic resonance imaging should be avoided in these cases, as this can be problematic for magnetic foreign bodies. A 2006 article asserts that pupil dilation is important for thorough ophthalmoscopy, and the risk of precipitating acute angle closure glaucoma, even with routine use, is outweighed by the benefit of a complete examination [10]. For patients with ocular trauma, dilation is important for evaluating suspected intraocular FBs. In our case, dilation allowed prompt visualization of the intraocular FB hidden behind the iris and ensured timely treatment to preserve the patient’s vision.

## Conclusions

In clinical practice, the occurrence of intralenticular FB remains a rarity, presenting significant diagnostic challenges for cases with a lack of introacular inflammation and identification of FB. The importance of accurate diagnoses and the removal of FB is emphasized by the potential risk of visual conditions following prolonged retainment of metallic FB's, such as endophthalmitis, cataract, retinal detachment, and siderosis bulbi. Our unique case alerts clinicians to the utility of dilation for better lens visualization.
